# Account of Hytatids in a Herniary Sac

**Published:** 1809-04

**Authors:** John Taunton

**Affiliations:** Surgeon to the City and Finsbury Dispensaries, and City Truss Society; Lecturer on Anatomy, Surgery, and Physiology. Greville Street, Hatton Garden


### *Account of Hytatids in a Herniary Sac.*

MRS. ANN TURNER, ætat. 67, has been visited several times in the last two years by Mr. Jackson, for symptoms resembling those which arise from strangulated hernia; but the existence of that disease was never made known till after the attack came on, which terminated fatally.

November 30, 1808, the patient complained of a tightness across the umbilical region; great pain over the abdomen, with hiccough and vomiting. These symptoms had on former occasions frequently yielded to purgative remedies, which were now prescribed, but without effect.

December 1. Passed a very restless night, and was much worse in every respect. This day, for the first time, she mentioned a swelling in the groin. Some draughts, each containing 25 drops of tincture of opium, were given, but not retained on the stomach; an enema composed of an infusion of nicotiani was injected, and attempts were made to reduce the hernia, but without effect.

I first saw her at 9 o'clock in the evening, when she appeared very low, but the hiccough returned only when she attempted to swallow; the pain on the abdomen was not so great as on the preceding day; the pulse was regular, moderately full, and did not exceed 90. The hernia was under Poupart's ligament, on the inside of the femoral vessels. It was small, and on continued pressure, receded under the ligament between the lower edge of the external oblique and transoersalis muscles; this circumstance has occasionally misled inexperienced practitioners, one fatal instance of which I have related in another place.

She had had this complaint for several years, but could not state when it had been reduced, as it did not appear to her to have been of the least consequence, and she never had noticed it with much attention; neither could we make her believe that her present complaint arose from that small swelling; however, she consented, with considerable reluctance, to have the operation performed early in the morning, provided the symptoms continued.

2nd. 4 A. M. She appeared much the same as on the preceding evening; but her countenance, and in some instances, incoherent answers, afforded an unfavourable prognosis.

On dividing the integuments, cellular and adipose substance, with the fascia, a small tumour of a rough unequal surface was exposed; the contents were evidently fluid. On its being opened, about an ounce of a limpid fluid escaped from a sac, which proved to be an hydatid. At the posterior part of this *sac* was seated the hernial sac, forming a tumour not larger than a chesnut, but adhering firmly in *every part* to the surface of the contained intestine, so as to render its separation wholly impossible; the sac was returned with the intestine. After the contracted part at the neck had been carefully divided by a longitudinal incision, one suture was passed through the integuments, and the edges of the wound supported by straps of adhesive plaster. From these difficulties the operation took more time than is usually required, but she scarcely expressed a sense of pain. The pulse was full, and did not exceed 80.

Small doses of magn. vit. in aq. am. acet. et aq. mentha sat. were ordered to be taken frequently during the day.

3d. P. M. Every unfavourable symptom had subsided; the medicine and some broth had been retained on the stomach, and a gentle perspiration was diffused over the body. The medicines were ordered to be continued, and a purging clyster to be injected.

3d. 5. A. M. She had slept for several hours during the night, and had not had any return of either the hiccough or sickness, but no evacuation by stool. Notwithstanding the cessation of pain, and that the nourishment taken was retained in the stomach, and though she had slept, she was evidently sinking.

1. P. M. Quite composed and sensible, but sinking fast; she died at 5 P. M. 33 hours after operation.

On examining the part by dissection, the hydatid was found to adhere to the anterior part of the true peritoneal herniary sac, which from being very small, and the adhesions not permitting the enlargement of the hydatid, was entirely covered by it.

The "rough, irregular" appearance on the outside of the sac, appeared to be produced by the adhesion of the remains of some hydatids, which had burst within the large one.

The intestine within the sac was inflamed, but only a small part of the circumference was included within the stricture, so that the canal was preserved even at the diseased part, of sufficient size to admit a bougie the size of a large finger.

The intestine above the stricture was slightly inflamed, and rather distended with flatus; below, it was contracted without any appearance of inflammation.

The fatal termination of this disease when only a small part of the circumference of the gut is included in the sac, has been noticed by the late Mr. Joseph Else in a case in St. Thomas's Hospital, which was mistaken for an enlarged gland.* The two cases are also similar, in proving that stools could not be procured, although the intestinal canal was pervious in each. I have also observed the same circumstances to occur in omental hernia; though Mr. Charles Bell, in his work on Operative Surgery, has given an opposite opinion. I am, &c.

JOHN TAUNTON,

Surgeon to the City and Finsbury Dispensaries, and City Truss Society; Lecturer on Anatomy, Surgery, and Physiology.

*Greville Street, Hatton Garden, Sept.* 20, 1808.

---

* See Medical Observations and Enquiries, vol. 4, p. 355.

